# Fall Prediction and Prevention Systems: Recent Trends, Challenges, and Future Research Directions

**DOI:** 10.3390/s17112509

**Published:** 2017-11-01

**Authors:** Ramesh Rajagopalan, Irene Litvan, Tzyy-Ping Jung

**Affiliations:** 1 School of Engineering, University of St. Thomas, St. Paul, MN 55105, USA; 2 Department of Neurosciences, University of California, San Diego, CA 92093, USA; ilitvan@ucsd.edu; 3 Institute for Neural Computation, University of California, San Diego, CA 92093, USA; jung@sccn.ucsd.edu

**Keywords:** fall prediction, fall prevention, internet of things, information fusion, wearable and ambient sensing

## Abstract

Fall prediction is a multifaceted problem that involves complex interactions between physiological, behavioral, and environmental factors. Existing fall detection and prediction systems mainly focus on physiological factors such as gait, vision, and cognition, and do not address the multifactorial nature of falls. In addition, these systems lack efficient user interfaces and feedback for preventing future falls. Recent advances in internet of things (IoT) and mobile technologies offer ample opportunities for integrating contextual information about patient behavior and environment along with physiological health data for predicting falls. This article reviews the state-of-the-art in fall detection and prediction systems. It also describes the challenges, limitations, and future directions in the design and implementation of effective fall prediction and prevention systems.

## 1. Introduction

Falls are a major cause of injuries, with over one-third of older adults experiencing at least one fall or more each year [1]. Fall injuries are among the 20 most expensive medical conditions. For instance, in 2015, costs for falls to Medicare alone totaled over $31 billion [1]. In addition, the average hospital cost for a fall injury is over $30,000. Hence, there is a critical need for the development of cost-effective fall prediction systems to reduce the financial and health burdens associated with the consequences of a fall. Fear of falling has been shown to be associated with negative consequences such as avoidance of activities of daily living, less physical activity, falling, depression, and lower quality of life [2]. The effect of fall detection units on the fear of falling has been studied by Brownsel et al. [3]. Their study showed that persons who wore the fall detector reported more confidence and independence in their daily activities, and considered that the detector improved their safety. Their study concluded that the fear of falling is affected by user perception of the reliability and accuracy of the fall detector.

Fall-related medical care incurs a high financial expenditure with a $43.8 billion projected cost by 2020 [4]. Existing systems mainly focus on detecting a fall with little emphasis on fall prediction and prevention. Hence, there is an urgent need for developing monitoring systems that can minimize this cost and improve the quality of life for persons who suffer from falls. Fall prediction and prevention systems are of utmost importance to accomplish this task and can help reduce the financial, physical, and emotional consequences of a fall. However, fall prediction is a challenging problem due to the combination of intrinsic and extrinsic fall risk factors that contribute to a fall. Intrinsic factors include age, fall history, mobility impairments, sleep disturbances, and neurological disorders. Extrinsic factors include slippery surfaces, improper footwear, poor lighting, and clutter. A comprehensive fall prediction system should capture the interaction between these risk factors for effective fall risk assessment. There are very few comprehensive reviews on fall detection, prediction, and prevention systems. These reviews mainly focus on falls in the context of wearable sensors, gait analysis, assistive devices, signal processing, and machine learning algorithms [5,6,7,8]. However, they do not address the challenges inherent in the multifactorial nature of falls, extrinsic fall risk factors, user-centric design principles, and performance analysis in real life conditions on frequent fallers. Our work addresses these gaps by presenting a comprehensive review of the challenges in fall prediction and prevention systems along with potential future research directions. While fall detection and prediction systems are both aimed at reducing the consequences of a fall using various sensors and algorithms, there are some key differences:

- Fall detection systems alert the user and healthcare provider after a fall has occurred to expedite and improve the medical care provided. These systems are aimed at identifying different kinds of falls: falls from walking or standing, falls from standing on supports, e.g., ladders etc., falls from sleeping or lying in the bed and falls from sitting on a chair. These systems often use threshold-based algorithms to detect falls. The performance metrics of fall detection systems include precision (true positive rate), specificity (true negative rate), and false positive rate.
- Fall prediction systems are aimed at alerting the subjects before the occurrence of a fall thus preventing the emotional and health consequences of a fall. These systems should identify all scenarios and circumstances leading to a fall and provide a framework to predict them. This framework must be constructed based on data acquired from various scenarios surrounding fall-related events. Information on fall-related events is usually collected through questionnaires, fall diaries, and phone calls. This information is often augmented with data collected from various sensors to improve reliability and accuracy.
- Fall prediction systems must capture the multifactorial nature of falls for reliable fall risk estimation. These include environmental, physiological, and psychological risk factors. Fall detection systems mainly focus on physiological risk factors such as gait, mobility, and vision.
- Fall detection systems usually focus on developing a fall detection device using wearable sensors that can be integrated into watches, shoes, belts, etc. Fall prediction systems focus on information fusion from both wearable and ambient sensors for reliable estimation of fall risk. These systems also include the design and evaluation of user interfaces such as smartphone applications for fall prevention intervention and educating subjects on fall risk factors.

Wearable health systems based on wireless body sensor networks (BSNs) are becoming increasingly popular for real time biomedical monitoring in static or mobile scenarios [9]. A comprehensive survey of BSNs is presented by Chen et al. [10]. The main objective of BSNs is to enable pervasive monitoring of physical activities and behaviors, as well as physiological and biochemical parameters of the patients during their daily activities. The most commonly measured vital signs include: ECG, heart rate, blood pressure, blood oxygen saturation (SpO_2_), core/surface body temperature, posture, and physical activities. Vital signs are regularly collected and remotely monitored by medical professionals achieving a more autonomous caretaking system. In such systems, on body sensors are wirelessly connected via a multi hop network to a dedicated sink node such as a tablet or a smart phone. Sensors are worn by the subject in various forms such as shoes, eyeglasses, earring, clothing, gloves and a watch. Spatio-temporal variation in gait is a reliable indicator of fall risk, however, existing systems focus on gait analysis in clinical settings and are not geared towards continuous gait analysis in a home environment. Wearable BSNs can be used to address this challenge. For example, in gait analysis using BSNs, motion sensors are worn or attached to various parts of the patient’s body, such as the foot and waist. These sensors, which may be accelerometers, gyrosensors, force sensors, or strain gauges can measure various characteristics of the human gait. Signal processing algorithms and feature extraction techniques can be developed to process various gait features such as stride speed, stride length, and inter leg spacing for predicting a future fall.

In this article, we describe the recent trends, challenges, and limitations in designing effective fall prediction and prevention systems. We present recommendations for overcoming these limitations and describe key focus areas for future research.

## 2. Current Work and Limitations

Fall detection and prediction systems can be broadly categorized into two types: context-aware systems and wearable devices. Context-aware systems use ambient sensors such as cameras, pressure sensors, vibration sensors, and infrared sensors to classify activities of daily living and predict falls [5].

### 2.1. Wearable Fall Detection and Prediction Systems

Wearable systems use sensors such as accelerometers and gyroscopes for gait analysis and mobility monitoring [11]. Recent research on wearable technologies for fall detection and prediction has primarily focused on fusing data from accelerometers and gyroscopes for fall risk assessment [12,13,14,15,16,17]. Bourke et al. [18] developed a fall detection system and analyzed various combinations of acceleration magnitude, sensor velocity, and body posture. Their work concluded that a fusion of all three features gives the highest fall sensitivity and the lowest false positive rate when using a triaxial accelerometer. Bianchi et al. [19] recently tested a waist-mounted wearable sensor system composed of an accelerometer and a barometric pressure sensor. They tested a variety of fall scenarios both indoors and outdoors with the objective of reducing false alarms. Their results showed that adding a barometric pressure sensor may prevent false positives under common scenarios of use. However, similar to many studies reporting simulated falls, their work was only tested on healthy young adults.

Comfort of wearable sensors is very important in fall detection and prediction systems since it entails long-term continuous use. A recent study reported that in a three-month home trial of case enclosed waist-mounted accelerometer among aging adults, subjects transferred the wearable device between various body locations due to bruising and discomfort [20]. Hence, an important requirement for wearable systems includes device size and comfort, not to cause bruising or discomfort over time, even if attached continuously in the same location.

Howcroft et al. [21] presented a comprehensive analysis of fall-risk prediction using two types of wearable sensors (accelerometers, pressure-sensing insoles), four accelerometer locations (head, pelvis, left and right shank), and three types of models (neural network, support vector machine, naïve Bayesian). Their work concluded that multi-sensor gait assessments provided the best input data for fall-risk prediction, using a combination of posterior pelvis, head, and left shank accelerometers and a neural network. The best single-sensor model used a posterior pelvis accelerometer, dual-task gait data, and a neural network.

Sannino et al. [22] developed an approach that obtains data through a tag placed on the subject’s chest. They perform windowing of the data to classify windows as being part of fall or non-fall actions, and a final window composition to assess whether or not each global action was a fall. Their approach was tested on real-world data consisting of fall and non-fall events. Their results are promising and provide a strong foundation for implementation of real-world fall detection systems. Fortina and Gravina [23] developed a novel real-time non-invasive fall detection and alarm notification system using a wearable accelerometer and a smart phone. Their system is able to trigger fall events using different alerting modalities enabling prompt emergency interventions. The experimental results on 20 subjects demonstrated a 97% sensitivity, 83% specificity, and 90% precision. This system provides a benchmark for the design and evaluation of future fall detection systems.

However, there is no research on developing unobtrusive wearable devices for constant measurement of blood pressure to detect orthostatic hypotension and the associated fall risk. A recent survey indicates that 32% of users stop using wearable sensors after six months and 50% slightly after a year [20]. Hence new research is needed to investigate the characteristics of wearable systems such as obtrusiveness, cost, and user friendliness to improve their appeal among older adults.

### 2.2. Ambient Sensors for Fall Detection and Prediction

Camera-based sensors have been widely used in fall detection and prediction systems [24,25]. In such systems, multiple cameras are used to monitor the daily activities of persons in their home environment. Although camera-based systems provide detailed information on certain fall risk factors, they suffer from several drawbacks such as privacy, cost, and user acceptance. Another major drawback of such systems is their inability to track persons outside of the camera’s range of visibility.

Proximity sensors are another example of ambient sensors used for detecting falls. Hirata et al. [26] developed a method for controlling a passive intelligent walker to prevent the user’s fall according to the support polygon and the walking characteristic of the user. The proximity sensors are attached to a walking aid device for measuring sudden changes in the movements and distance of a person from the proximity sensors. Such sensors have a short proximity range and a higher false alarm rate since a person stepping away from the walker can be misinterpreted as a fall. Bian et al. [27] developed a robust fall detection approach by analyzing the tracked key joints of the human body using a single depth camera. The proposed fall detection approach uses an infra-red based depth camera that can operate in dark environments. However, their approach cannot detect falls ending lying on the furniture. Hilbe et al. [28] proposed a “Bed-exit” alarm for preventing bedside falls. Their system uses pressure sensors that were integrated on the side rails of the patient’s bed to track their attempt to get out of bed. If the pressure sensor value exceeds a certain threshold, an alarm is sent to clinicians in order to prevent a fall from occurring.

Commercially available ambient sensors are often used to interact with falls prevention exercise games. For example, Pisan et al. [29] and Kayama et al. [30] developed systems that utilize Microsoft Kinect sensor with a game developed for older adults at risk of falling. The game measures changes to patients’ functional and cognitive abilities by performing physical and cognitive tasks simultaneously, as multi-tasking is known to be a predictive factor for future falls.

Clinical fall risk assessments often involve questionnaires and functional assessments of posture, gait, cognition, and other fall risk factors [31]. These clinical assessments provide a snapshot overview of fall risks, but are often subjective, and use threshold assessment scores to classify people as fallers and non-fallers [32,33]. However, geriatric fall risk should be more accurately modeled using a continuum and multiple risk categories, such as low, moderate, and high fall risk. Longitudinal monitoring of aging adults in a free-living environment using unobtrusive sensors and mobile health tools can provide a more accurate and objective assessment of fall risk.

Shany et al. described the use of wearable sensors and methodologies for fall risk assessment in supervised and unsupervised environments [34]. However, they did not discuss testing and validation of fall risk assessment methodologies and real-world implementations on frequent fallers. Tong et al. proposed a hidden Markov model-based method using a tri-axial accelerometer for predicting falls [35]. However, their method was not tested on real-world falls and aging adults who are frequent fallers.

Table 1 shows a qualitative summary of various wearable and ambient sensor-based fall detection and prediction systems. The three major research gaps in existing fall prediction systems can be summarized as follows:

- Lack of a comprehensive fall prediction system. Existing systems do not address information fusion that captures contextual and physiological data from wearable and ambient sensors for fall risk estimation.
- Dearth of user-friendly interfaces and feedback techniques to actively engage and empower patients towards effective techniques to prevent falls.
- No efficient web interfaces to help clinicians visualize health data and assess fall risk.

## 3. Fall Prevention Systems

**Figure 1:**
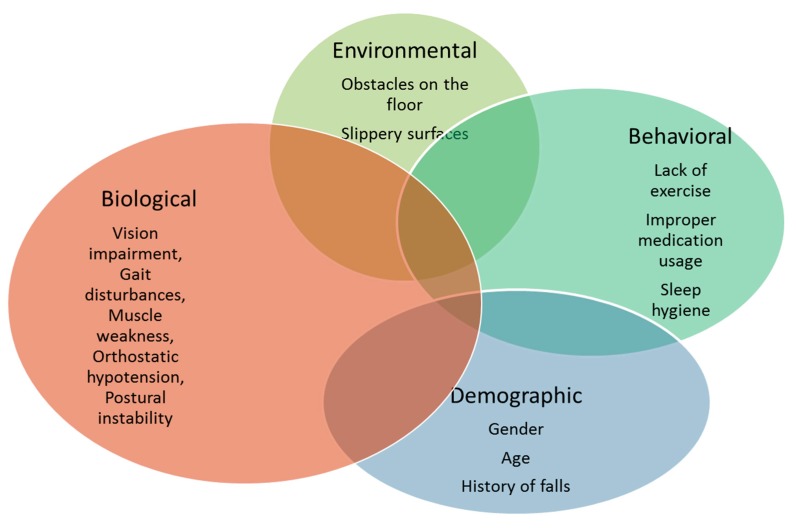
Interaction between various fall risk factors.

Fall prediction involves the design of signal-processing techniques and machine-learning algorithms for reliable estimation of fall risk and providing timely alerts before the occurrence of a fall. Fall prevention is focused on the design and implementation of techniques and intervention programs for mitigating fall risk factors, improving gait and mobility, and preventing a future fall. Examples of fall prevention intervention techniques include exercise, medication review, and home modification. Both fall prediction and prevention are complex multifactorial problems due to the interaction between physiological, behavioral, and environmental factors that contribute to a fall. Fall risk assessment is an important technique that identifies intrinsic (gait, muscle weakness, neurological or visual deficits, etc.) and extrinsic (poor lighting, inappropriate footwear, etc.) risk factors that help determine the most appropriate interventions for effective fall prevention. Figure 1 shows the various fall risk factors and the complex interactions between them. For instance, a combination of gait impairment, improper medication usage, muscle weakness, and slippery surfaces can substantially increase the risk of falling.

### 3.1. Current Work

This section describes current work and limitations of fall prevention systems. Existing fall prevention systems mainly focus on rehabilitation robotics and wearable devices to assist patients with gait impairments and prevent future falls. Di et al. developed a cane type assistive device that helps aging adults to safely avoid environmental hazards and prevent falls [36]. Their system uses the center of gravity of the user in conjunction with the cane sensors to determine fall risk. Majumber et al. designed a fall prediction system using a smart phone and smart shoe [37]. The pressure sensors on the shoe along with accelerometer in the smart phone is used to collect gait and balance information. Their system uses a smart phone application that triggers an alarm when gait abnormalities are detected. Future work should focus on information fusion from both wearable and ambient sensors for assessing a variety of fall risk factors such as vision and sleep disturbances, medications, and environmental hazards.

A popular approach to falls prevention involves methods targeting the restoration of muscle strength and balance for prevention of fall risks [38,39]. Exercise interventions are becoming an increasingly popular approach for fall prevention and there is extensive literature supporting the effectiveness of these interventions in reducing falls and the risk of falling [40]. There are a wide range of falls prevention intervention systems focused on overcoming falls and reducing the risk of falling. Pre-falls prevention intervention systems (Pre-FPIs) focus on monitoring and supporting patients who have not yet experienced a fall, but may be considered to be at risk of falling. These systems support the delivery of targeted physical activities and educational programs that increase awareness of fall risks and help develop strategies to identify and overcome environmental fall hazards. Pre-FPIs aim to overcome intrinsic fall risk factors such as vision, balance, muscle strength and cognitive decline [41,42]. With regard to intrinsic fall risk factors, functional ability was the main focus of a number of studies [43,44,45]. In these studies, various technologies were used to proactively mitigate observed deficits in functional ability. For example, Visvanathan et al. [42] developed a wearable sensing system that monitors the physical activity of patients who are hospitalized and considered to be at a high risk of falling as a result of functional decline. De Morais and Wickstrom [43] developed a game based technology using tai chi, to help improve the stability of subjects with balance impairments and impaired mobility. Initially, subjects were given a demonstration of pre-recorded tai chi activities at the start of the game and are required to mimic those movements during gameplay.

Post-fall prevention intervention systems (Post-FPIs) focus on developing technologies for persons who have already experienced a fall and deliver interventions to reduce the future occurrence of a fall [46]. Post-FPI supports the delivery of re-active interventions. These systems involve a diagnostic assessment function, whereby the cause of the fall, which triggered the post fall intervention, is identified along with other fall risk factors.

Cross fall prevention intervention systems (CFPIs) are technologies that support and deliver a combination of pre-fall, post-fall and fall injury prevention interventions. Shi et al. [47] developed a CFPI that uses a smart-phone application for assessing fall risks. Their approach also uses traditional clinical tests and detects falls after they have occurred in order to prevent fall-related injuries. Another example of CFPI is that of Silva et al. [48]. In their approach, older adults are assessed for intrinsic fall risks and are provided an exercise regime to reduce those intrinsic risks such as functional decline and a decline in muscle strength.

### 3.2. Fall Prevention Intervention

Intervention techniques used for preventing fall risks are typically administered either by clinicians or self-administered by patients [49,50,51,52]. Physical activities are effective intervention techniques used to mitigate intrinsic fall risk factors. Recent studies have demonstrated that virtual reality and gaming technologies are interactive and effective for patients to engage in exercise activity compared with more traditional approaches [49,50,51,52]. For example, Chao et al. [53] have investigated the barriers that lead to a lack of adherence to falls rehabilitation exercises and issues concerning older adults’ behavior towards exercising. Their results showed that the application of the self-efficacy theory to enhance exercise behavior to engage older adults in physical activities increases adherence rates of exercise programs.

Post-FPI intervention techniques consist of functional assessment, cognitive assessment, and environmental assessment. Functional assessment is the main intervention technique that is widely used to determine intrinsic fall risk factors such as functional ability deficits [54,55,56,57,58]. For instance, Majumder et al. [59] developed a smart-phone-based fall risk assessment system to monitor abnormal gait patterns of older adults performing physical activities. The gait patterns were collected from users over a period of time while performing activities of daily living (ADL) such as walking. Staranowicz et al. [60] developed a system that monitors the walking patterns of older adults during their ADLs at home. Their approach identifies functional decline using an autonomous robot. The systems proposed in [61,62,63] use both cognitive assessment and functional assessment techniques to assess functional ability deficits, balance and cognitive impairments. In these studies, patients perform physical activities and cognitively demanding tasks to determine fall risks. Du et al. [64] developed a robotic system that screens the subjects’ home, for environmental fall hazards. This system is operated remotely by clinicians, to automate home assessments that are typically conducted by in-person visits.

## 4. Challenges

The design of fall prediction and prevention systems faces several significant challenges. These are described below.

### 4.1. Performance in Real-Life Conditions

Fall prediction and prevention systems need to be accurate, reliable, robust, and cost-effective. High specificity and sensitivity are the main goals of a reliable fall prediction system. This is sometimes achieved in experimental environments, but when applied to a real world setting, the performance is often unknown. Fall detection devices are designed and tested under controlled conditions, for example they use data from falls and activities of daily living of young adults. These experiments are simulated at the discretion of authors due to the lack of a standardized procedure or a public database for comparison. Only few studies incorporate data from older people [65,66], although their participation is limited to perform a set of simulated activities of daily living for a few minutes or hours. It is evident that existing systems have been mainly tested in laboratory environments with controlled conditions and do not include frequent fallers and aging adults as test subjects. However, falls are more common among older adults with a history of falls and patients with neurological disorders such as advanced Parkinson’s disease or even more common at early stages in atypical parkinsonian disorders such as Progressive Supranuclear Palsy [67]. Hence, future work should focus on longitudinal studies of fall detection and prediction systems in real life conditions on a diverse group that includes frequent fallers, aging adults, and persons with neurological disorders. Future work on IoT-enabled fall detection and prediction systems should address the long term assessment of comfort level and obtrusiveness of the wearable devices among older adults.

### 4.2. User-Centric Design

Fall prediction systems frequently use an IoT-enabled approach for pervasive monitoring. IoT enabled monitoring systems combine data from multiple sensors and transmit data wirelessly to a smart phone for pervasive monitoring and fall prediction. Such systems should incorporate user feedback and preference throughout the monitoring process. Existing monitoring systems mainly track and report data from wearable sensors and do not engage the users in the monitoring process. Recent surveys have shown that wearable technology enabled systems for falls have little appeal among aging adults due to lack of feedback and user engagement [68]. User acceptance poses a major challenge since older adults may not be familiar with wearable devices and mobile health technologies. Recent work has focused on developing smartphone-based systems and mobile applications (apps) for fall detection and prediction [69]. However, these studies have not tested the usability and acceptability of the mobile apps among aging adults.

### 4.3. Security and Privacy

Privacy, integrity, and confidentiality of data are major concerns in fall prediction systems that process and transmit sensitive information about patient health. Context-aware systems and vision-based systems are much more prone to privacy concerns compared to wearable devices such as accelerometers and gyroscopes. Privacy issues pose a huge challenge in testing fall prediction systems in free living environments and community settings. This is particularly challenging in IoT-enabled systems where patient sensitive health data are stored in smartphones and transmitted over wireless networks that are vulnerable to attacks. Recent work has focused on mobile cloud based systems in health care for data storage and processing to protect patient privacy [70]. However, such systems have not been adopted for fall prediction. A comprehensive fall prediction system should include an interdisciplinary approach that fosters a synergy between engineers, computer scientists, and healthcare professionals to ensure compliance with HIPPA regulations and standards on patient confidentiality.

The adoption of a cloud computing IoT paradigm provides several benefits for data security and privacy in the context of healthcare services and IoT-enabled fall prediction systems. Identity privacy is an important concern in cloud based IoT systems. The technique of pseudonyms has been widely adopted to address identity privacy, but the periodically updated pseudonyms and certificates lead to intolerable computational cost for resource-constrained sensors. Location privacy is especially critical in IoT-enabled fall prediction systems, since the frequently exposed location privacy would disclose the living habit of the user. The widely adopted technique in cloud based IoT systems is to hide the location through pseudonyms. However, since the location information is not directly protected, it cannot resist dynamic tracing attacks. Zhou et al. [71] proposed a new efficient privacy preserving data aggregation technique that addresses identity and location privacy. However, their work was only tested on a single user and further research is needed to test the efficiency of their techniques on multiple users.

### 4.4. Energy Optimization

IoT-enabled fall prediction and prevention systems should incorporate energy-optimization techniques for conserving the battery power of sensors. A limited energy budget is the primary constraint on smartphones and wearable sensors. To address this issue, future work should develop energy-optimization techniques such as power gating, sampling frequency scaling, and configurable operational modes to conserve the battery power of sensors [72]. Compressive sensing is an efficient technique for data acquisition and conserves energy by reducing the amount of wireless data transmission. Smartphone-based systems should address the storage and computational limitations of the smartphone to process Big data due to the limited battery power. In addition, mobile technologies such as smartphone apps that provide patient feedback may perform differently depending on the smartphone model in which they are installed. This possibility should be investigated for successful implementation of mobile health technologies in real-world settings such as assisted living facilities.

Cloud computing is particularly useful in IoT-enabled fall detection and prediction systems that often use battery constrained smartphones for implementing information fusion and machine learning algorithms. The adoption of cloud computing paradigm enables the execution of secure multimedia-based health services, thus eliminating the need for executing computationally intense multimedia and security algorithms on mobile devices with limited computational capacity and battery power. Cloud computing provides a flexible storage and processing infrastructure for performing both online and offline analyses of large volumes of sensor data. However, achieving energy efficiency in both data transmission and processing is still an open research issue [73]. Techniques such as data caching mechanisms for reusing collected data in time-tolerant applications and middleware for compressing data in continuous and long-duration monitoring scenarios can be used to address this challenge.

## 5. Recommendations and Future Work

The multifactorial nature of falls warrants a comprehensive interdisciplinary approach for effective fall prediction and prevention. Future research should focus on the following key areas.

### 5.1. Information Fusion and Machine Learning

Contextual information about patient behavioral patterns and environment play a crucial role in predicting falls. For instance, significant deviations in sleep patterns and medications, as well as gait abnormalities can indicate an underlying medical concern that may increase fall risk. Hence reliable estimation of fall risk necessitates information fusion from wearable and ambient sensors and a decision support system for meaningful inferences. Gravina et al. [74] have presented a comprehensive and systematic review of the state-of-the-art techniques on multi-sensor data fusion in BSNs. The survey provides a systematic categorization and common comparison framework of the literature. They have identified distinctive properties and parameters affecting fusion design choices at various levels (data-level, feature-level, and decision-level) of fusion techniques. Future work should focus on developing context aware BSNs and information fusion techniques that are capable of adapting to different contexts by extracting and transferring knowledge from one context to another.

Machine-learning algorithms such as hidden Markov models and decision trees can be used to detect anomalies in patient behavior that may indicate an impending fall. Machine-learning algorithms can also be used to correlate predictive variables with specific fall risk factors such as gait impairments, muscle weakness, reduced flexibility, and orthostatic hypotension. This would improve the clinical value of fall risk assessment, allowing clinicians to identify specific factors that increase fall risk and design customized interventions.

Multiple heterogeneous sensors commonly used in fall detection and prediction systems introduce several sources of uncertainty. In many cases, we might have sensors that are simply not working, or that are giving incorrect readings. More generally, a given sensor will have a specific signal to noise ratio, and the types of noise that are corrupting the signal might also vary. Future work should focus on developing a principled and unifying framework for quantifying sensor uncertainty in ways that can represent and process uncertain values. Bayesian graphical models that directly incorporate sensor noise coupled with efficient inference algorithms can be used to address sensor uncertainty [75]. Machine learning algorithms and data mining techniques should be contextualized and placed within a wide body of health-related background knowledge related to various fall risks. The operating context of these techniques will vary from training to deployment and between residents and households. Hence, design and implementation of new methods that are robust to these variations are critical. Continuous streams of sensor data should be mined for temporal patterns that vary between individuals. These temporal patterns can be directly built into the model-based framework for learning context sensitive and specific patterns at both group-wide and individual levels.

### 5.2. User Interfaces for Providing Feedback to Clinicians and Patients

With the proliferation of smartphones, fall detection and prediction can be improved by developing user-friendly mobile apps for engaging patients and clinicians in the monitoring process. Mobile apps that can track user behavior and provide feedback about fall risk factors such as sleep, exercise, gait, and medication intake can help in preventing future falls. However, a user-centric approach is essential for sensor data to be meaningful to the user. Experts suggest that displaying a large amount of raw sensor data can lead to cognitive overload and user discouragement. Hence, mobile app design should adopt a user-centric approach where user feedback is solicited and incorporated at various stages of the app design. This will ensure end-user adoption and engagement while promoting a sustained behavior change. In particular, future work should focus on testing the usability and acceptability of mobile apps among older adults who are at frequent risk of falling. Usability testing should assess technical effectiveness and efficiency of various tasks on the app. Examples include metrics for measuring the time required to complete various tasks, tasks that require assistance, and errors that occur during navigation.

Future work should also focus on developing mobile interfaces for clinicians that will support their decision-making process and provide clinically relevant information. For instance, apps and modules can be designed to provide timely evaluation of information about patient physiological health, gait, activity, and sleep patterns. The information repository will help clinicians assess the severity of fall risk and provide tailored feedback to patients.

### 5.3. Smart Phone Based Fall Detection and Prediction

The evaluation of fall detection and prediction approaches has almost exclusively focused on the accuracy of the detection algorithm. However, if a smartphone is employed as a central element of the architecture, a specific assessment of the performance of the smartphone platform should be considered. Owing to the restricted battery capacity and computing power of most smart phones, the consumption of energy and computing resources of these devices when employed in fall detection and prediction applications must be carefully examined. Future work should investigate the coexistence of fall detection mobile apps with other conventional apps and functionalities such as text messaging, web browsing, etc. The impact of other resource consuming apps on the performance of fall detectors and predictors should be thoroughly analyzed [76]. Techniques such as reducing the sensor sampling frequency, cloud computing, and running the app in the background can effectively conserve the battery power. In particular, the performance assessment and benefits of a cloud computing paradigm in IoT-enabled fall prediction systems is an important area for future research.

### 5.4. Environmental Fall Risk Factors

Extrinsic fall risk factors are most often overlooked in fall prediction and prevention systems. Hence, we need new technologies to assist patients in their efforts to reduce fall risk due to extrinsic fall risk factors. Examples of such factors include environmental hazards such as poor lighting, wet floor surfaces, loose rugs, clutter, and poorly organized furniture. Effective fall prevention should incorporate proactive approaches for the delivery of targeted activities and education programs for overcoming environmental fall risk hazards. It has been demonstrated that gaming applications are widely accepted by aging adults for promoting targeted exercises [77]. Hence, fall prevention systems will benefit from interactive 3D gaming applications that can simulate a variety of environmental hazards that might occur at patients’ home. The gaming applications will promote the awareness of extrinsic fall risk factors and provide strategies for preventing a future fall.

### 5.5. Comparison of IoT-Enabled Systems with Clinical Systems

To demonstrate the utility of an IoT-enabled approach for fall risk assessments, future research should focus on a comparison between IoT-enabled fall risk assessment and clinical assessments in relation to prospective fall occurrences. This would determine the advantages of an IoT-enabled fall prediction system and demonstrate if they can provide better accuracy compared to clinical assessments. Researchers should also investigate whether a combination of sensor-based and clinical fall risk assessment would optimize fall prediction, and if specific risk factors can be better identified using sensors. While some work has been done comparing IoT-enabled fall risk assessment to clinical assessments [78,79], additional research is required to conclusively demonstrate the advantages of IoT-enabled systems for fall prediction.

### 5.6. Biomedical Signal Based Fall Prediction

Falls are a debilitating and expensive problem for many patients with Parkinson’s disease (PD). Persons with PD are twice as likely to fall as people with other neurological conditions [67]. Freezing of gait (FOG) is one of the most common causes of falls and is very often the most distressing symptom in persons with PD. Relatively few studies have investigated the detection and prediction of FOG using electromyographic (EMG) and electroencephalographic (EEG) signals [80,81]. Some studies reported using an EMG pattern to detect the onset of FOG [82,83]. Handojoseno et al. [80] developed a neural network based classifier for early detection of FOG in PD patients using EEG signals. Complemented with special treatment such as sensory cuing, this classification system could be used in helping patients with FOG to ‘unfreeze’ this symptom before it affects the gait leading to falls. Their approach could identify the onset of freezing in PD patients during walking with sensitivity and specificity of 80%. Although these results are encouraging, future work should focus on developing signal processing algorithms for utilizing EEG and EMG signals for a more accurate prediction of FOG. Different aspects of the EEG signal, when combined, may provide more significant information, leading to a better classification accuracy for predicting FOG. Future research should also investigate dimension reduction of EEG features, further exploration regarding the location of electrodes, and developing new classification methods for improving both sensitivity and specificity.

Surface electromyography (sEMG) sensors measure the electrical potentials generated by muscle activity using noninvasive electrodes offering an inherent advantage in predicting movements and obtaining a shorter computational time. Studies have found that sEMG signals could be successfully applied to gesture recognition, gait analysis, and limb prosthetic control [84,85]. Recent research on using sEMG sensors for fall detection applications have shown promising results. Xi et al. [86] developed feature extraction and pattern recognition methods using sEMG for daily living activities monitoring and fall detection. The results of their study demonstrated that a system with four sEMG sensors was sufficient for achieving a sensitivity and specificity of 90% with less than 10% misclassifications. Leone et al. [87] presented a study of a real-time and low invasive sEMG-based system for the assessment of fall risk. They developed a simple and real time threshold-based approach for classification of fall risk. Future work should focus on increasing the robustness and performance of the system. Cheng et al. [88] developed a framework for investigating the feasibility of fall detection based on sEMG and accelerometers. Their approach demonstrated activity recognition accuracy of over 98%, demonstrating the feasibility of the proposed method in daily activities awareness. However, their methods were tested on healthy subjects and did not include aging adults and frequent fallers.

### 5.7. Accuracy of Fall Prediction

Future research should focus on the design of new techniques, clinical assessments, and algorithms for improving the accuracy of fall prediction systems. Despite the relatively high prevalence of falls especially in people with PD, accurate methods for predicting a future fall, especially during the early stages of the disease, remain elusive. The utility of a variety of clinical balance tests for predicting falls has been studied. Balance assessments including the Berg Balance Scale (BBS) [89], the Timed Up and Go (TUG) [90], the Functional Gait Assessment (FGA) [91], and recently developed Balance Evaluation Systems Test (BESTest) [92] still demonstrate a clinically relevant proportion of false-positive and false-negative predictions. Duncan et al. [93] analyzed the ability of four balance assessment techniques to predict falls in persons with PD over six and twelve month periods. Their study demonstrated that a six-month follow-up resulted in greater accuracy of fall prediction compared to a twelve-month follow-up.

Future work should focus on robust determination of the accuracy of fall prediction for older adults. Although the older population is considered to be the most at risk, our review has shown that very few studies have been conducted to determine fall prediction accuracy in older adults. For example, the Hidden Markov model based algorithm developed by Tong et al. [35] can predict falls 200~400 ms before the collision, and can distinguish fall events from other daily life activities with 100% sensitivity and 100% specificity. However, their results are based on the data from simulated falls of young healthy subjects, and are not applicable for older adults who are most susceptible to fall. Moreover, falls have not been studied in patients who have multiple causes of falls such as those with multiple system atrophy. Falls in such patients can occur due to orthostatic hypotension leading to syncopal events, FOG, imbalance or cardiovascular diseases occurring in the aging population. Also, future work should use large samples of older patients in different acute care settings to determine sensitivity and specificity with higher precision in different clinical and patient conditions.

## 6. Conclusions

This article surveys the state-of-the-art in fall prediction and prevention systems. We describe recent trends, limitations, challenges, and future research directions for designing IoT-enabled fall prediction systems. Fall prediction is a complex multifactorial problem that involves interaction between physiological, environmental, and behavioral risk factors. Our review indicates that existing fall detection and prediction systems are mainly tested in laboratory environments and do not capture the interactions between various fall risk factors. The main challenges in designing effective fall prediction systems include evaluating performance among frequent fallers and aging adults, user-centric design, security and privacy in data transmission and storage, and energy optimization. We present recommendations for future work in the design of efficient fall prevention systems. Main focus areas for future research include: development of unobtrusive wearable devices for constant measurement of blood pressure; data fusion from wearable and ambient sensors, user interface design, assessment of external fall risk factors, and comparisons to clinical fall risk assessments. In particular, new technologies are needed that can reliably identify external fall risk factors such as environmental hazards and deliver targeted educational interventions for preventing falls. Significant advances in these research areas will eliminate several barriers that exist for the widespread adoption of IoT-enabled fall prediction and prevention systems by health care providers, patients, and clinicians.

## Figures and Tables

**Table 1:** Qualitative comparison of various fall detection and prediction systems.

Article	Sensor	Subjects	Obtrusive	Comments
Bourke et al. [18]	Waist mounted accelerometer	10 young healthy subjects and 10 elderly heathy subjects	medium	Threshold based algorithm achieves 100% specificity and sensitivity with a false-positive rate of less than 1 false-positive (0.6 false-positives) per day of waking hours.
Binachi et al. [19]	Waist-mounted wearable sensor system composed of an accelerometer and a barometric pressure sensor.	20 young healthy volunteers	medium	The proposed system demonstrated an accuracy, sensitivity and specificity of 96.9%, 97.5%, and 96.5%, respectively, in the indoor environment, with no false positives generated during extended testing during activities of daily living.
Howcraft et al. [21]	Accelerometers and pressure sensing insoles	75 individuals who reported six month prospective fall occurrence	Low	The best performing fall prediction system used a neural network, dual-task gait data, and input parameters from head, pelvis, and left shank accelerometers.
Sannino et al. [22]	A tag placed on subjects’ chest	real-world database containing a set of fall and non-fall actions	Low	Their method achieves better accuracy than four state of the art machine learning algorithms.
Hirata et al. [26]	Proximity sensors attached to walking aid device	unspecified	Low	Their method uses a passive intelligent walker to prevent the user’s fall according to the support polygon and the walking characteristic of the user.
Bian et al. [27]	Infrared camera	Four healthy subjects	Medium	The proposed approach uses the infra-red based depth camera that can operate in dark environments. Experimental results show that the accuracy of the proposed algorithm is improved by 11.8% compared with a state-of-the-art fall detection algorithm.
Hilbi et al. [28]	Pressure sensors	Older adults in a hospital	Low	The results show that sensitivity and the specificity of the proposed approach are 96% and 99% respectively indicating a satisfactory performance. Further consistently designed studies such as randomized controlled trails are required to show the effect of bed-exit alarm systems on fall risk.
Pisan et al. [29]	Microsoft Kinect camera	57 elderly patients	Medium	Their results show that for users who are at risk of falling, the slowing down in reaction time due to cognitive load is much larger than for users who are not at risk of falling.
